# Optimization of Lung Surfactant Coating of siRNA Polyplexes for Pulmonary Delivery

**DOI:** 10.1007/s11095-022-03443-3

**Published:** 2022-11-29

**Authors:** Domizia Baldassi, Thi My Hanh Ngo, Olivia M. Merkel

**Affiliations:** Department of Pharmacy, Pharmaceutical Technology and Biopharmaceutics, Ludwig-Maximilians University of Munich, Butenandtstraße 5, 81377 Munich, Germany

**Keywords:** air-liquid interface, polyplexes, pulmonary delivery, pulmonary surfactant, siRNA delivery

## Abstract

**Purpose:**

The aim of this study was to understand how coating with a pulmonary surfactant, namely Alveofact, affects the physicochemical parameters as well as *in vitro* behavior of polyethylenimine (PEI) polyplexes for pulmonary siRNA delivery.

**Methods:**

Alveofact-coated polyplexes were prepared at different Alveofact:PEI coating ratios and analyzed in terms of size, PDI and zeta potential as well as morphology by transmission electron microscopy. The biological behavior was evaluated in a lung epithelial cell line regarding cell viability, cellular uptake via flow cytometry and gene downregulation by qRT-PCR. Furthermore, a 3D ALI culture model was established to test the mucus diffusion and cellular uptake by confocal microscopy as well as gene silencing activity by qRT-PCR.

**Results:**

After optimizing the coating process by testing different Alveofact:PEI coating ratios, a formulation with suitable parameters for lung delivery was obtained. In lung epithelial cells, Alveofact-coated polyplexes were well tolerated and internalized. Furthermore, the coating improved the siRNA-mediated gene silencing efficiency. Alveofact-coated polyplexes were then tested on a 3D air-liquid interface (ALI) culture model that, by expressing tight junctions and secreting mucus, resembles important traits of the lung epithelium. Here, we identified the optimal Alveofact:PEI coating ratio to achieve diffusion through the mucus layer while retaining gene silencing activity. Interestingly, the latter underlined the importance of establishing appropriate *in vitro* models to achieve more consistent results that better predict the *in vivo* activity.

**Conclusion:**

The addition of a coating with pulmonary surfactant to polymeric cationic polyplexes represents a valuable formulation strategy to improve local delivery of siRNA to the lungs.

**Graphical Abstract:**

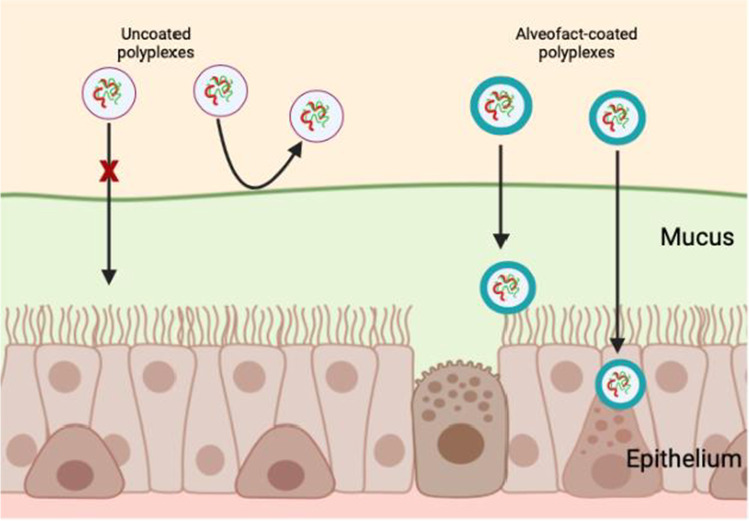

**Supplementary Information:**

The online version contains supplementary material available at 10.1007/s11095-022-03443-3.

## Introduction

The recent authorization of the first mRNA vaccines for the prophylaxis of COVID-19 has shed the light on the advantages of RNA-based therapeutics as potential treatment for a variety of diseases. Besides the mRNA vaccines, in the last four years we have witnessed the approval of four siRNA therapies [1, 2]. RNA interference, in fact, can theoretically be tuned to downregulate any target sequence, whether endogenously or exogenously produced [3]. Although the currently approved siRNA drugs are administered intravenously and target the liver, research efforts are focused on the development of delivery systems that can target tissues beyond the liver and that are designed for local administration [4, 5]. Particularly pulmonary administration appears as a desirable route of delivery for siRNA. Due to its large surface area, low enzymatic activity and ease of access, the development of formulations for direct administration to the lung appears as a promising strategy [6]. Furthermore, the development of an siRNA therapy for direct administration to the airways could be beneficial for treating several pathological conditions affecting the lung for which no curing treatment is available yet, such as cystic fibrosis, asthma, acute lung injury, lung cancer but also viral infections as in the case of the recent SARS-CoV-2 outbreak [7–11]. Although two siRNA formulations reached clinical trials for intranasal administration, no formulation for direct administration to the lungs has been approved yet [12]. Despite the undeniable benefits offered by pulmonary administration, some major obstacles must be overcome to reach the target site as well as an efficient downregulation. Branching of the airways, mucus secretion and mucociliary clearance represent indeed crucial barriers hampering the activity of siRNA. For this reason, suitable delivery systems that can overcome the hurdles of the lung should be developed [13]. The nanocarrier, in fact, should not only protect the payload from degradation, but also diffuse through the mucus layer typical for the airways, particularly in the diseased state [14]. While the upper airways are covered by a mucus layer rich in lipids and glycoproteins, particularly mucin, the lower tract is covered by a thin layer of lung surfactant. Lung surfactant is secreted by alveolar type II cells and is responsible for reducing surface tension as well as for first line defense against external intruders [15]. It is composed mainly of lipids such as phosphatidylcholine, phosphatidylglycerol and cholesterol, which account for about 90% of the total mass. The remaining 10% consists of proteins, to which the surfactant specific hydrophilic proteins SP-A and SP-B belong that play a role in the innate immune and inflammatory response, and the hydrophobic SP-B and SP-C proteins, which help exerting the biophysical function of the lung surfactant. Pulmonary surfactant represents in fact the first biological fluid encountered by the delivery system when reaching the deep lung and it forms a biomolecular corona around the nanoparticles that can alter biodistribution, cellular uptake and cytotoxicity of the nanoparticles [16]. Although pulmonary surfactant can be considered an obstacle for delivering siRNA to the deep lung, previous studies suggest that it could represent an ally indeed [17]. Notably, pulmonary surfactant coating of polymeric delivery systems was reported to have a beneficial effect on siRNA delivery of different systems, such as PLGA-based nanoparticles [18] or dextran nanogels [19]. Additional studies also suggested that lung surfactant did not negatively influence the transfection efficiency of polymer-based delivery systems, while lipid-based delivery systems were in fact negatively affected [20]. On this basis, we decided to repurpose a broadly studied cationic polymer for siRNA delivery, polyethylenimine, with the addition of Alveofact coating, a commercially available pulmonary surfactant, following the formation of PEI/siRNA polyplexes. Although cationic polymers can efficiently condense and deliver siRNA to the cells, limitations are generally observed in terms of inadequate endosomal escape as well as high toxicity linked to the cationic nature of the polymer [21, 22]. In contrast, coating with pulmonary surfactant was shown to improve the safety as well gene silencing profile of non-viral delivery systems for siRNA [23]. Therefore, we have established a method for coating PEI polyplexes with Alveofact pulmonary surfactant at different PEI:Alveofact coating ratios. We tested the different formulations in terms of physicochemical behavior, stability and *in vitro* activity to identify the most promising one. We observed that Alveofact coating improved the gene silencing activity in comparison to uncoated polyplexes in a lung epithelial cell line. To further investigate the polyplexes in a more relevant *in vitro* setting, we developed an air-liquid interface culture of the respiratory tract that retains tight junctions as well as mucus secretion. ALI cultures represent a valid tool to reproduce some of the main features of the healthy as well as diseased respiratory tract *in vitro* and can be thus considered a more suitable instrument to test drug delivery systems for pulmonary delivery [24]. After testing Alveofact-coated polyplexes at ALI, we identified a formulation able to penetrate the mucus layer as well as to efficiently downregulate the expression of an endogenously expressed housekeeping gene. The experiment underlined the importance of testing delivery systems in appropriate *in vitro* models that better predict the *in vivo* behavior of the formulation. The resulting formulation is considered an efficient strategy to improve the delivery of siRNA to lung epithelial cells particularly in disease conditions accompanied with a deficiency of endogenous pulmonary surfactant, such as in patients suffering from acute respiratory distress syndrome (ARDS), or even in CARDS resulting from severe course of COVID-19 infection [25]. In this regard, Alveofact coating not only improved the transfection efficiency, but also helped drug spreading and absorption after pulmonary administration to more distal lung regions and thus lead to better therapeutic outcomes.

## Materials and Methods

### Materials

HEPES (4-(2-hydroxyethyl)–1-piperazineethanesulfonic acid), PEI 25 kDa, heparin sodium salt, paraformaldehyde solution, FluorSave™ Reagent, Eagle’s Minimum Essential Medium (EMEM), RPMI-1640 Medium, fetal bovine serum (FBS), Penicillin-Streptomycin solution, Dulbecco’s Phosphate Buffered Saline (PBS), trypsin-EDTA solution, 200 mM L-glutamine solution, Paraformaldehyde, Tween20, agarose and Alcian Blue solution (1% in 3% acetic acid pH 2.5) were purchased from Sigma-Aldrich (Darmstadt, Germany). Lipofectamine 2000, SYBR gold dye, AF488-anti-rabbit secondary antibody, rhodamine phalloidin, 4′,6–diamidino–2-phenylindole dihydrochloride (DAPI), Alexa Fluor™ 647 NHS ester and Alexa Fluor™ 488 NHS ester were obtained from Life technologies (Carlsbad, California, USA). Transwell® polyester membrane cell culture inserts (0.4 μm pore size) were purchased from Corning (New York, USA). PneumaCult ALI differentiation medium, hydrocortisone and heparin were purchased from Stemcell Technologies (Vancouver, Canada). Alveofact was purchased from Lyomark Pharma (Oberhaching, Germany). ROTI®GelStain Red and bovine serum albumin were purchased from Carl Roth GmbH (Karlsruhe, Germany). Dicer substrate double-stranded siRNA (DsiRNA) targeting human GAPDH, non-specific DsiRNA and amine-modified siRNA were purchased from integrated DNA Technologies (Leuven, Belgium).

### Preparation of Alveofact-Coated Polyplexes

Alveofact-coated polyplexes were formed by first preparing dilutions of PEI 10 kDa in RNase free water at a concentration of 1 mg/ml. Stocks of polymer and siRNA were further diluted in 10 mM HEPES buffer pH 7.4 to reach the desired concentration. The polymer dilution was added to the siRNA dilution and incubated for 20 min to obtain polyplexes at a defined N/P ratio of 6. In the meantime, different dilutions of Alveofact were prepared for Alveofact:PEI ratios (w/w) of 0, 1:5, 1:2.5, 1:1, 2.5:1, 5:1, 10:1, followed by sonication in a water bath without heating for 20 min. Two stocks of Alveofact were used, 0.2 mg/ml and 2 mg/ml, to keep the volume for each formulation constant. Alveofact:PEI ratio of 0 equals to no Alveofact added. Once the polyplex incubation time was completed, polyplexes were gently mixed with post-sonicated solutions of Alveofact and incubated for 10 min. The forming particles were subsequently subjected to a second sonication step for 20 min in the water bath to establish the Alveofact outer coating.

### Characterization of Polyplexes

#### Size, Polydispersity Index and Zeta (ζ) Potential of Alveofact-Coated Polyplexes

Hydrodynamic size and polydispersity index (PDI) were measured by dynamic light scattering using a Zetasizer Nano ZS (Malvern Instruments, Malvern, UK). Polyplexes were prepared with siRNA in 10 mM HEPES pH 7.4 and 100 μl were added to a disposable microcuvette for analysis. Measurements were performed at 173° backscatter angle running 10 runs three times per sample. Results are shown as average size (± SD). For ζ-potential measurements, the samples were further diluted to 700 μl with 10 mM HEPES buffer pH 7.4 and added to a folded capillary cell for ζ-potential measurement, which were analyzed by Laser Doppler Anemometry (LDA). A total of three runs per sample was performed, with each run consisting of 30–50 scans. Results are shown as mV ± SD.

#### TEM

The morphology of uncoated and coated polyplexes was analyzed at transmission electron microscopy. Briefly, 3.5 μl of freshly prepared polyplexes were applied to pre-coated Quantifoil holey carbon supported grids and negatively stained using 2% uranyl acetate. Micrographs were digitally recorded on a Tecnai G2 Spirit TEM at 120 kV. Data was collected under low dose conditions at a nominal magnification of 90,000 X and a nominal defocus of – 0.9 μm using an TVIPS XF216 2048 × 2048 pixel CCD camera (TVIPS, Gauting, Germany).

#### SYBR Gold

SYBR Gold Assay was used to assess the percentage of free siRNA in the formulations after production of Alveofact-coated polyplexes produced by different coating methods. Alveofact-coated polyplexes were prepared at N/P 6 with 100 pmol siRNA at different Alveofact:PEI ratios (w/w). Of each polyplex suspension, 100 μL of was added to a white FluoroNunc 96-well plate. Subsequently, 30 μL of a 4X SYBR Gold solution was added to each well, and the plate was incubated for 10 min in the dark. Fluorescence was measured on a FLUOstar OMEGA plate reader (BMG Labtech, Ortenberg, Germany) using a 492 and 555 nm excitation and emission wavelength, respectively. Free siRNA was used as 100% value. Measurements were carried out in triplicate, and the results were shown as mean value ± SD (*n* = 3).

#### Release Study

Stability of polyplexes is influenced by the presence of anions in biological fluids and cell culture medium containing serum. Therefore, heparin, a polyanion that potentially competes with nucleotides, was used to investigate the release capacity of siRNA from polyplexes. Alveofact-coated polyplexes were prepared in HEPES 10 mM pH 7.4 at N/P 6 with 100 pmol at different Alveofact:PEI ratio (w/w). Heparin was dissolved in HEPES 10 mM pH 7.4 to obtain the concentration of 0.2 USP units/μL, followed by 2-fold serial dilutions. Aliquots of 100 μL of each polyplexes solution were added to a white FluoroNunc 96-well plate with subsequent addition of 10 μL of heparin at different concentrations (0.125, 0.25, 0.5, 1, 2 USP units/well). After 30 min of incubation, 30 μL of a 4X SYBR Gold solution was added to each well and the plate was incubated for 10 min in absence of light. Fluorescence determination and free siRNA calculation were performed similarly to SYBR Gold Assay as described above. Measurements were executed in triplicate, and results were shown as mean value ± SD (*n* = 3).

#### Gel Integrity Assay

To confirm the integrity of Alveofact-coated polyplexes after sonication, a gel retardation assay was performed. A 1% Agarose gel was prepared and stained with ROTI®GelStain Red. Polyplexes were prepared with 300 pmol siRNA at three different Alveofact:PEI ratios (0, 2.5:1 and 5:1). As positive control, polyplexes were treated with 1 USP unit of heparin. 3 μL of low range ssRNA ladder (New England BioLabs, Ipswich, Massachusetts, USA) and 3 μL of siRNA were respectively diluted in 27 μL of RNA free water. 30 μL of each sample were mixed with 5 μL of loading dye (New England BioLabs, Ipswich, Massachusetts, USA), loaded into the slots of a gel, and electrophoresis was run at constant voltage of 200 V for 15 min in Tris-borate EDTA buffer. The gel was visualized using a ChemiDoc MP imaging system (Bio Rad, Hercules, California, USA).

### Polyplexes Stability in Storage Condition

To evaluate the stability of Alveofact-coated polyplexes, batches at different coating conditions were prepared and stored at room temperature protected from light. At specific time points (0, 24, 48, 72, 96 and 168 h), hydrodynamic size was measured by dynamic light scattering. Briefly, 100 μl were added to a disposable microcuvette and measurements were performed at 173° backscatter angle performing 10 runs three times per sample. Results are shown as average size (± SD).

### Polyplexes Stability in Presence of Mucin

Stability of polyplexes in presence of mucin was evaluated by gel retardation assay. Briefly, a 1% Agarose gel stained with ROTI®GelStain Red was prepared as well as polyplexes loaded with 200 pmol siRNA at two Alveofact:PEI ratios (2.5:1 and 5:1). Two stock solutions of mucin were prepared at two different concentrations, 3 and 6 mg/mL, to achieve final mucin solutions of 1 mg/mL and 2 mg/mL respectively after the addition of polyplexes. 20 μL of each formulation was mixed with either 10 μL of HEPES 10 mM pH 7.4, 10 μL of mucin 3 mg/mL or 10 μL of mucin 6 mg/mL and incubated for 30 minutes. As positive controls, 2 USP units of heparin were subsequently added to samples containing 20 μL polyplexes and 10 μL of mucin 6 mg/mL. After incubation, each sample was mixed with 5 μL of loading dye, loaded into gel and electrophoresis was run at 200 V for 15 min in Tris-borate EDTA buffer solution. The gel was visualized using a ChemiDoc MP imaging system (Bio Rad, Hercules, California, USA).

### Cell Culture

The human non-small carcinoma cell line H1299 was cultured in RMPI-1640 medium supplemented with 10% FBS and 1% P/S. 16HBE14o- cells were grown in EMEM medium supplemented with 10% FBS and 1% P/S. Cells were passaged every 3 days with trypsin 0.25% and subcultured in 75 cm^2^ flasks. Cells were maintained in a humidified atmosphere at 37°C and 5% CO_2_.

### Cellular Uptake by Flow Cytometry

To evaluate the cellular uptake of Alveofact-coated polyplexes, amine-modified siRNA was labeled with succinimidyl ester (NHS) AlexaFluor488 fluorescent dye according to the manufacturer’s protocol. The resulting AF488-siRNA was then purified via ethanol precipitation and spin column as previously described [26]. H1299 cells were seeded at a density of 50.000 cells/well in 500 μl medium and incubated for 24 h at 37°C and 5% CO_2_. The day after, cells were transfected with polyplexes prepared at different Alveofact:PEI coating ratios (0, 1:5, 1:2.5, 1:1, 2.5:1, 5:1) with 50 pmol AF488-siRNA. Positive controls consisted of Lipofectamine 2000 lipoplexes, whereas untreated cells and samples treated with free siRNA were used as negative controls. Cells were incubated for 24 h at 37°C and 5% CO_2_. Cells were then harvested, washed in PBS and resuspended in PBS/2 mM EDTA for analysis via flow cytometer (Attune NxT, Thermo Fisher Scientific, Waltham, Massachusetts, USA) for the median fluorescence intensity (MFI) of AF488-siRNA using 488 nm excitation and a 530/30 nm bandpass emission filter. Samples were gated by morphology based on forwards/sideward scattering with a minimum of 10.000 viable cells. Results are displayed as mean values ± SD.

### *In Vitro* GAPDH Gene Knockdown

For gene silencing experiments, 100.000 16HBE14o- cells were seeded in a 12-well-plate in 1 ml medium and were incubated for 24 h at 37°C and 5% CO_2_. The day after, cells were transfected with 100 μl of polyplexes prepared at different Alveofact:PEI coating ratios (0, 1:5, 1:2.5, 1:1, 2.5:1, 5:1) with 100 pmol of GAPDH or scrambled siRNA. Positive controls consisted of Lipofectamine 2000 lipoplexes, while negative controls consisted of untreated cells. After 24 h, cells were harvested and processed to isolate RNA using the PureLink RNA mini kit according to the manufacturer’s protocol (Life technologies, Carlsbad, USA) with additional DNase digestion. Afterwards, cDNA was synthesized from total RNA using the high-capacity cDNA synthesis kit (Applied Biosystems, Waltham, Massachusetts, USA). The obtained cDNA was then diluted 1:10 in water and amplified on QuantStudio 3 Real-Time PCR (Thermo Fisher Scientific, Waltham, Massachusetts, USA) using the SYBR™ Green PCR Master Mix (Thermo Fisher Scientific, Waltham, Massachusetts, USA) with primers of human GAPDH (Qiagen, Hilden Germany) and β-actin (Qiagen, Hilden Germany). The RT-qPCR template consisted of an initial denaturation step for 10 min at 95°C, subsequently 40 cycles of 95°C for 15 s, annealing and elongation at 60°C for 1 min. Cycle threshold (Ct) values were obtained and GAPDH gene expression was normalized by corresponding β-Actin expression for each sample. The qPCR results were analyzed using the 2^−∆∆Ct^ method and presented as a relative quantity of transcripts. Values are given as mean values ± SEM.

### *In Vitro* Cell Viability

To evaluate the cell viability after incubation with Alveofact-coated polyplexes, an MTT assay was performed. 16HBE14o- cells were seeded at a density of 10.000 cells/well in 100 μl medium in a 96-well-plate. The day after, cells were then transfected with Alveofact-coated polyplexes at different Alveofact:PEI coating ratios (0, 1:5, 1:2.5, 1:1, 2.5:1, 5:1) containing 20 pmol scrambled siRNA and incubated for 24 h at 37°C and 5% CO_2_. Afterwards, medium was removed and replaced with 100 μl of a sterile 0.5 mg/ml 3-(4,5-dimethylthiazol-2-yl)-2,5-diphenyltetrazolium bromide (MTT) solution and incubated for 3 h at 37°C and 5% CO_2_. Medium was then removed and 200 μl DMSO was added to dissolve formazan crystals. Absorbance was read at 570 nm using a microplate reader (Tecan, Männedorf, Switzerland). Results are given as mean values of triplicates ± SD.

### Polyplexes Behavior in 16HBE14o- Cells Grown at ALI

#### 16HBE14o- Characterization under ALI Conditions

16HBE14o- cells were seeded at the density of 3 × 10^5^ cell/cm^2^ on the apical side of Transwell® polyester cell culture inserts (6.5 mm, 0.4 μm pore size) in 100 μl medium. The basolateral compartment was filled with 700 μl medium. After 72 h of incubation (day 3), medium was removed from the apical side while the medium on the basolateral side was replaced with PneumaCult™ ALI medium (Stemcell technologies, Vancouver, Canada) to obtain air-liquid interface conditions. Medium in the basolateral chamber was replaced every two days. To monitor the development of the polarized epithelial layer, transepithelial electrical resistance (TEER) was measured every day starting from day 1 after air-lift, using an EVOM epithelial volt/Ω meter (World Precision Instruments, Sarasota, USA). TEER values were corrected by subtracting the background of an empty Transwell® insert and medium. For the measurement, 200 μl and 700 μl of medium were added to the apical and basolateral side of the insert respectively, and TEER values were recorded using an STX2 electrode following the manufacturer’s instructions.

To evaluate the secretion of mucus by 16HBE14o- cells under ALI conditions, an alcian blue staining was performed. 7 days after air-lift, the cell layer was washed three times with PBS and fixed using 4% (v/v) paraformaldehyde for 15 min. Afterwards, the cell layer was washed again with PBS, incubated with 100 μL of alcian blue solution (1% in 3% acetic acid, pH 2.5) (Sigma-Aldrich) for 15 min and then washed again 3 times with PBS. The membrane was cut with a sharp point scalpel, mounted on glass slides using FluorSave™ reagent (Merck Millipore, Billerica, USA) and analyzed with a BZ-8100 (Biozero) fluorescence microscope (Keyence, Osaka, Japan).

To confirm the development of tight junctions under ALI conditions, the expression of zonula occludens protein-1 (ZO-1) was investigated by immunohistochemical staining. On day 7 after air-lift, the cell layer was washed 3 times with PBS and fixed in 4% paraformaldehyde for 15 min. After that, the cell layer was rinsed 3 times with PBS and permeabilized with 200 μL 0.3% Tween20 for 10 min. Afterwards, 200 μL of 5% BSA blocking buffer was added to the insert and incubated for 60 min. The membrane was then cut with a sharp point scalpel, placed in a 24-well-plate and incubated overnight with 300 μL of rabbit ZO-1 antibody solution (1:100 dilution in blocking buffer) at 4°C. On the following day, the membrane was washed 3 times with PBS and incubated with 300 μL of AF488 anti-rabbit secondary antibody (1:500 dilution in blocking buffer) for 60 min in the dark. The membrane was then washed 3 times with PBS and incubated with a 0.5 μg/ml 4′,6-diamidino-2-phenylindole (DAPI) solution for 15 min. Afterwards, the membrane was rinsed 3 times with PBS, mounted using FluorSave™ reagent on glass slides and analyzed with an SP8 inverted confocal scanning laser microscope (Leica Camera, Wetzlar, Germany). The images were exported from the Leica Image Analysis Suite and processed with the Fiji distribution of ImageJ.

#### Cell Uptake Study

To evaluate the cellular uptake of Alveofact-coated polyplexes in ALI culture, amine-modified siRNA was labelled with succynimidyl ester (NHS) modified AlexaFluo647 dye according to the manufacturer’s protocol and subsequently purified via ethanol purification as previously reported [26].

Differentiated 16HBE14o- cells were transfected with polyplexes prepared at different Alveofact:PEI coating ratios (0, 2.5:1, 5:1) with 100 pmol AF647-siRNA and incubated for 24 h at 37°C and 5% CO_2_. Afterwards, cells fixed in 4% PFA for 15 min, washed 3 times with PBS and permeabilized with PBS + 0.3% Tween20 for 10 min. Cytoskeleton was then stained by incubation with rhodamine phalloidin for 60 min, followed by nuclei staining with 0.5 μg/ml solution of 4′,6-diamidino-2-phenylindole (DAPI) for 15 min. The membrane was then cut and mounted using FluorSave™ reagent on a glass slide and analyzed with an SP8 inverted confocal scanning microscope (Leica Camera, Wetzlar, Germany). The images were exported from the Leica Image Analysis Suite and processed with the Fiji distribution of ImageJ.

#### Mucus Penetration Study

To test the ability of Alveofact-coated polyplexes to cross the mucus layer secreted by 16HBE14o- cells, cells were transfected with Alveofact-coated polyplexes at different Alveofact:PEI ratios (0, 2.5:1, 5:1) containing 100 pmol AF647-siRNA and incubated for 24 h at 37°C and 5% CO_2_. Once the incubation time was completed, AF488-wheat germ agglutinin was added to the cells and incubated for 15 min at 37°C and 5% CO_2_ to stain the mucus layer. Afterwards, cells were washed 2 times with PBS and the membrane was cut and mounted on glass slides using FluorSave™ reagent. Membranes were immediately analyzed with a SP8 inverted confocal laser scanning microscope (Leica Camera, Wetzlar, Germany). The images were exported from the Leica Image Analysis Suite and processed with the Fiji distribution of ImageJ.

#### GAPDH Knockdown in 16HBE14o- Cells at ALI

To measure the transfection efficiency of polyplexes in a mucus-presenting environment, 16HBE14o- cells grown at ALI conditions were transfected with Alveofact-coated polyplexes at different Alveofact:PEI ratios (0, 2.5:1, 5:1) containing 100 pmol GAPDH or scrambled siRNA and incubated for 24 h at 37°C and 5% CO_2_. Positive controls consisted of Lipofectamine2000 lipoplexes while negative controls consisted of blank/untreated cells. Once the incubation time was completed, cells were detached from the membranes and RNA was extracted using PureLink RNA mini kit (Life technologies, Carlsbad, USA) according to the manufacturer’s protocol. Samples were then processed for cDNA synthesis and qPCR as described above. Values are given as the mean of triplicates ± SEM.

### Statistics

Statistical analysis was performed with GraphPad Prism 5 software using One-Way ANOVA with Bonferroni post-hoc test, with p > 0.05 considered not significant (ns), and *p < 0.05. **p < 0.01, ***p < 0.005, **** *p* < 0.001 considered significantly different.

## Results and Discussion

### Physico-Chemical Characteristics of Alveofact-Coated Polyplexes

Size and surface charge of polyplexes regularly requires optimization to achieve efficient delivery to their target cells. In the case of pulmonary administration, the development of a delivery system able to deliver the payload to lung epithelial cells while penetrating the mucus barrier covering the epithelium is a prerequisite not only in the diseased state. In this regard, we aimed at developing Alveofact-coated polyplexes with optimized properties for pulmonary administration.

**Fig. 1:**
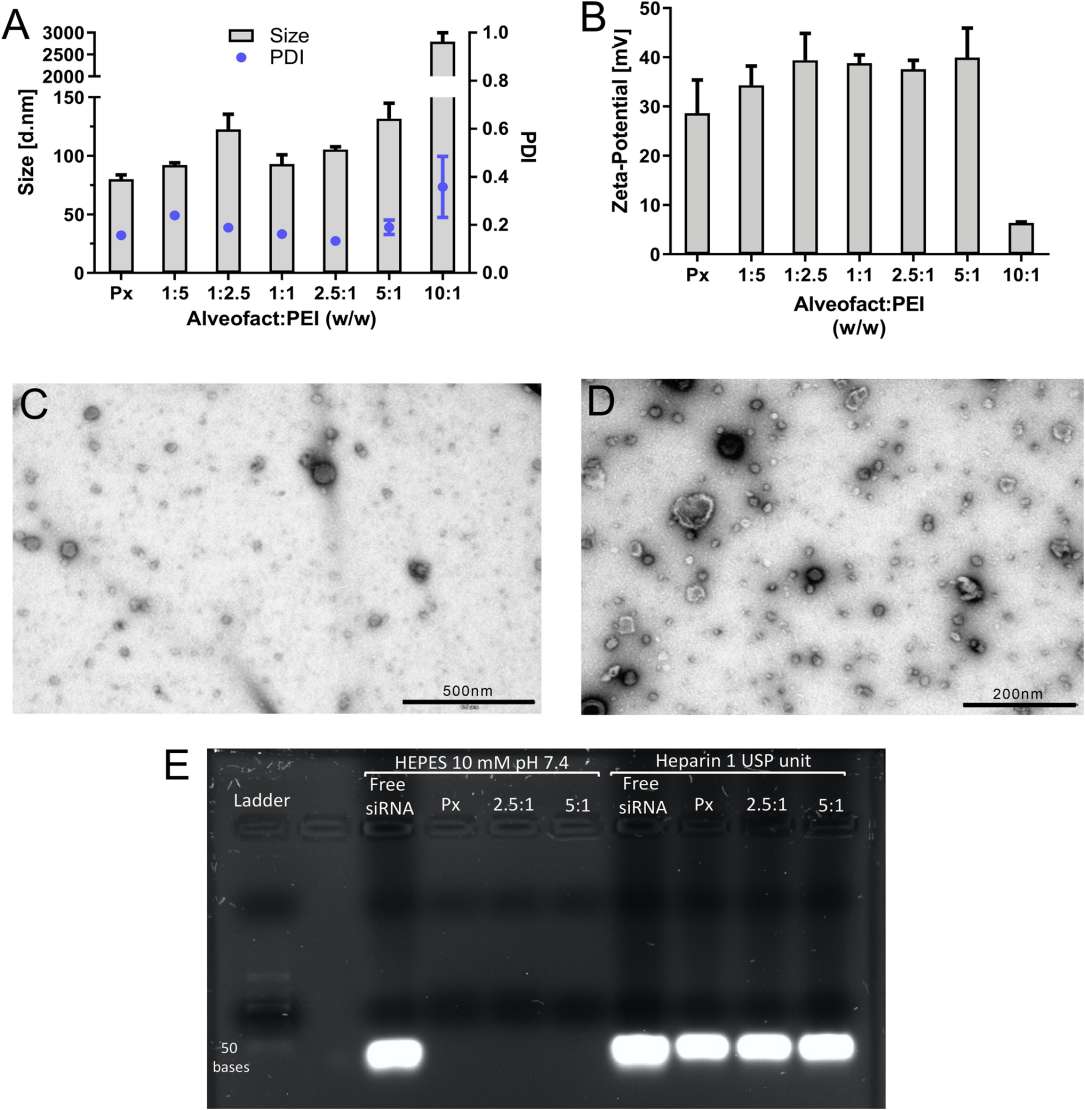
Physico-chemical properties of Alveofact-coated polyplexes. (**A**) Hydrodynamic diameter and polydispersity index, and (**B**) ζ-potential of Alveofact-coated polyplexes prepared at N/P 6 in HEPES 10 mM pH 7.4 at different Alveofact:PEI coating ratios. (**C**, **D**) TEM images of uncoated and Alveofact-coated and polyplexes, respectively. (**E**) Agarose gel electrophoresis for integrity tests of Alveofact-coated polyplexes prepared with 100 pmol siRNA. Positive controls consisted of free siRNA, uncoated polyplexes, and Alveofact-coated polyplexes (2.5:1 and 5:1) in 1 USP unit of heparin.

To achieve a successful coating of polyplexes, we included two sonication steps, a first one for Alveofact alone and a second one after adding Alveofact to siRNA/PEI polyplexes. In a preliminary formulation screening, we tried initially coated polyplexes with a single Alveofact sonication step prior to incubation with siRNA/PEI polyplexes. However, only polyplexes with unfavorable physicochemical properties were obtained (Supplementary Fig. 1). Conversely, the inclusion of a sonication step following incubation with lung surfactant resulted indeed in polyplexes with promising physicochemical parameters (Fig. 1A). An explanation for this observation could be the fact that Alveofact tends to self-assemble into multilamellar bodies and vesicles, leading to aggregation phenomena that prevent a homogeneous coating of polyplexes, consequently resulting in poor physicochemical parameters [27]. The inclusion of a sonication step seemed to favor the formation of smaller and more homogeneous surfactant vesicles, which are better incorporated in the hybrid delivery system [28]. First, we investigated the optimal Alveofact:PEI coating ratio required to achieve appropriate physicochemical characteristics. Polyplexes were prepared at different Alveofact:PEI coating ratios (0, 1:5, 1:2.5, 1:1, 2.5:1, 5:1, 10:1) and investigated in terms of size, PDI and ζ-potential. An N/P ratio of 6 was kept constant throughout the formulation study as it was previously shown to be ideal for pulmonary administration of siRNA/PEI polyplexes [29]. As presented in Fig. 1A, polyplexes prepared with a coating ratio between 1:5 and 5:1 showed desirable values in terms of size, PDI and ζ-potential. Sizes ranged from 90 to 120 nm, while PDI presented values around 0.2, similarly to uncoated polyplexes. However, polyplexes prepared at a coating ratio of 10:1 displayed extremely increased sizes and PDI as well as a decreased zeta-potential. We hypothesized that the excess of Alveofact used led to agglomeration phenomena, which caused loss of stability of the formulation. Furthermore, we observed that Alveofact coating did not influence the encapsulation efficiency of polyplexes. At N/P 6, only negligible siRNA release less than 0.3% of the encapsulated siRNA was detected for both coated and uncoated polyplexes (Supplementary Table 1). To confirm the presence of the Alveofact coating, TEM pictures were acquired for uncoated polyplexes and coated polyplexes at the representative Alveofact:PEI ratio of 2.5:1 (Fig. 1C, D). The pictures underlined a clear difference between coated and uncoated polyplexes. While Fig. 1C represents uncoated polyplexes as dark, homogenous, rounded dots, coated nanoparticles (Fig. 1D) present a lighter corona around the dark polymeric core, which can be assumed to be Alveofact coating. However, the surfactant layer is not as defined as the polymeric core, probably due to irregular coating of the polyplexes. The micrographs also reflect the presence of some empty vesicles, which could be a source of increased polydispersity. Nevertheless, the implementation of microfluidics could potentially help in the future to eliminate empty vesicles and to reduce polydispersity [30]. A similar experiment was performed by Mousseau *et al*., where a supported lipid bilayer from Curosurf was deposited onto silica nanoparticles [31]. The latter study resulted in comparable TEM images. However, while physico-chemical features of silica nanoparticles can be accurately tuned by synthesis, resulting in analogously spherical-shaped nanoparticles, polyplexes are more dynamic in terms of size, shape and morphology due to the fact that electrostatic interaction is the main driving force for polyplex formation.

Since a sonication step was included for preparing Alveofact-coated polyplexes, any detrimental effect of sonication on siRNA integrity was assessed by a gel integrity assay. In this experiment we tested the integrity of siRNA after sonication of uncoated polyplexes and two representative coated formulations (2.5:1 and 5:1). As positive controls, free siRNA and polyplexes incubated in presence of 1 USP unit of heparin were included in the gel (Fig. 1E), which was previously identified as the heparin concentration necessary to achieve a complete release of siRNA (Supplementary Fig. 2). The experiment confirmed the integrity of siRNA and complete encapsulation following sonication.

### Stability of Polyplexes

**Fig. 2:**
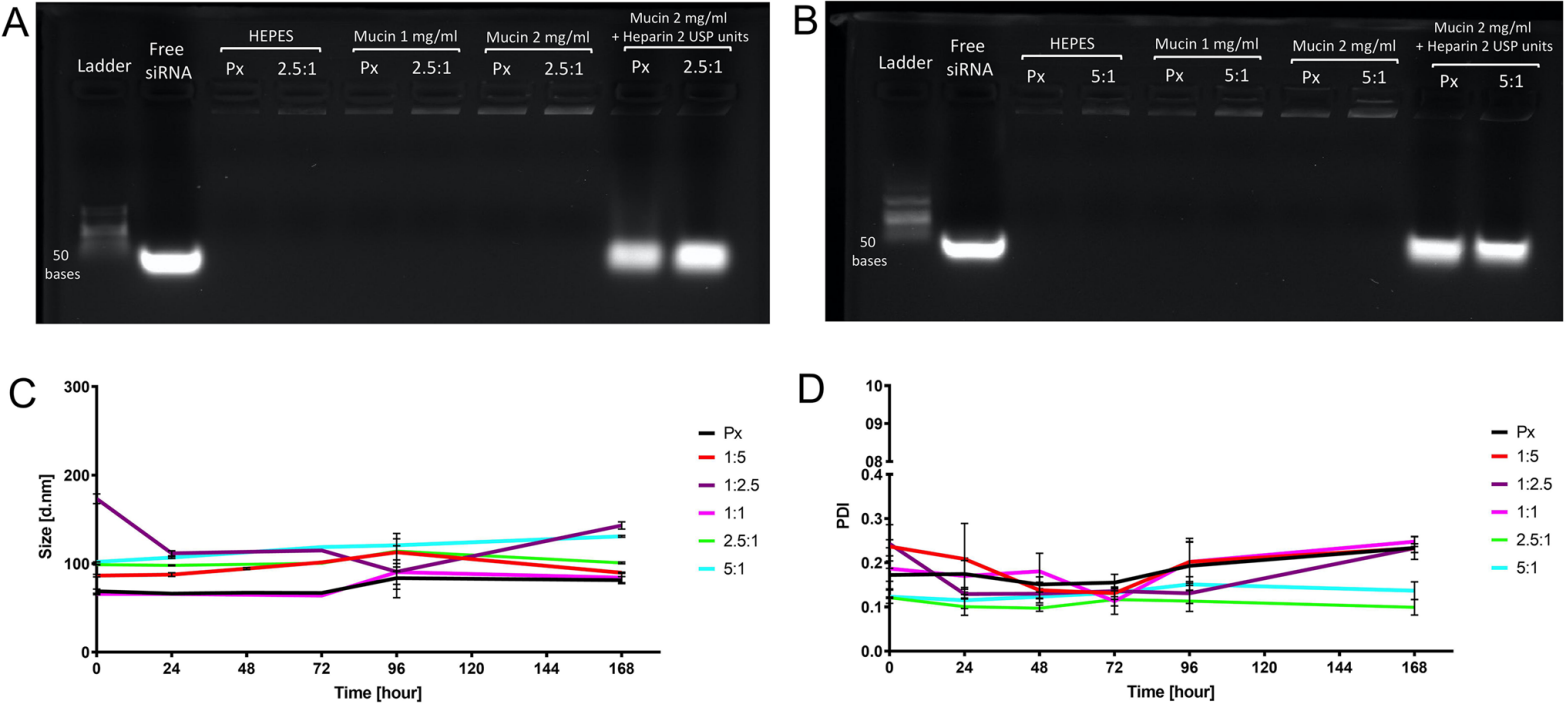
Stability of Alveofact-coated polyplexes. Agarose gel electrophoresis of Alveofact-coated polyplexes encapsulating 100 pmol of siRNA with Alveofact:PEI ratio of 2.5:1 (**A**) and 5:1 (**B**) in HEPES, mucin 1 mg/ml and mucin 2 mg/ml. Positive controls consisted of polyplexes in mucin 2 mg/ml and Heparin 2 USP units. (**C**, **D**) Hydrodynamic diameter and PDI of Alveofact polyplexes measured at 0 h, 24 h, 48 h, 72 h, 96 h, 168 h at room temperature with exclusion of light.

One of the main hurdles involved in local administration to the lungs is represented by the mucus barrier of the respiratory tract [32]. The mucus layer, especially in chronic obstructive diseases, has a strong impact on the stability of the formulation as well as on the efficient delivery of the cargo to the cells located below that layer. On this basis, we established a modified gel integrity assay to test the stability of Alveofact-coated polyplexes in presence of mucin, a negatively charged glycoprotein and one of the main components of pulmonary mucus (Fig. 2A, B). Due to its negative charge, mucin can potentially negatively impact the stability of polyplexes by replacing siRNA in the formation of the electrostatic interactions with the polymer. In this experiment, uncoated and coated polyplexes (2.5:1 and 5:1 Alveofact:PEI, which represent the coating ratios showing the best performance in terms of activity *in vitro*) were incubated at two different mucin concentrations. As positive controls, polyplexes were incubated with 2 USP heparin to obtain a full release of siRNA. Heparin and mucin are both negatively charged macromolecules or contain such macromolecules. In the reported experiments, heparin was used as a model molecule at concentrations high enough to disrupt polyplexes, a mechanism driven by the replacement of siRNA in the formation of the electrostatic interactions with the cationic PEI (Supplementary Fig. 2). Therefore, while the heparin concentration was intentionally used at a concentration able to disrupt polyplexes, for mucin a physiologically relevant and not an exaggerated concentration was selected to estimate stability of polyplexes in the lung.

From this experiment, we observed that no free siRNA was detected after incubation with mucin, thereby confirming the stability of polyplexes in presence of increasing concentrations of mucin. Moreover, Alveofact coating did not negatively affect the stability of the formulation in a physiologically relevant condition, confirming the suitability for pulmonary administration.

To assess the colloidal stability of polyplexes over time, the size of polyplexes prepared at different coating ratios was measured at different time points up to 1 week. As it can be observed from Fig. 2C, D, the formulations showed constant sizes and PDI over the entire period, with values ranging from 80 to 130 nm and 0.1–0.3, respectively. This experiment confirmed the stability of the formulation over a period of time suitable for formulation studies and excluded any negative influence of Alveofact coating on the stability of the formulation. The results are in line with previous studies suggesting that pulmonary surfactant coating improved the colloidal stability of polymer-based delivery systems and prevented release of siRNA in presence of competing polyanions such as mucin [19]. Further studies will be intended to investigate the stability of the formulation for longer times and to develop a spray dried powder for prolonged stability and inhalation based on our previously established spray-drying methodology for siRNA polyplexes [33].

### *In Vitro* Cellular Uptake

**Fig. 3:**
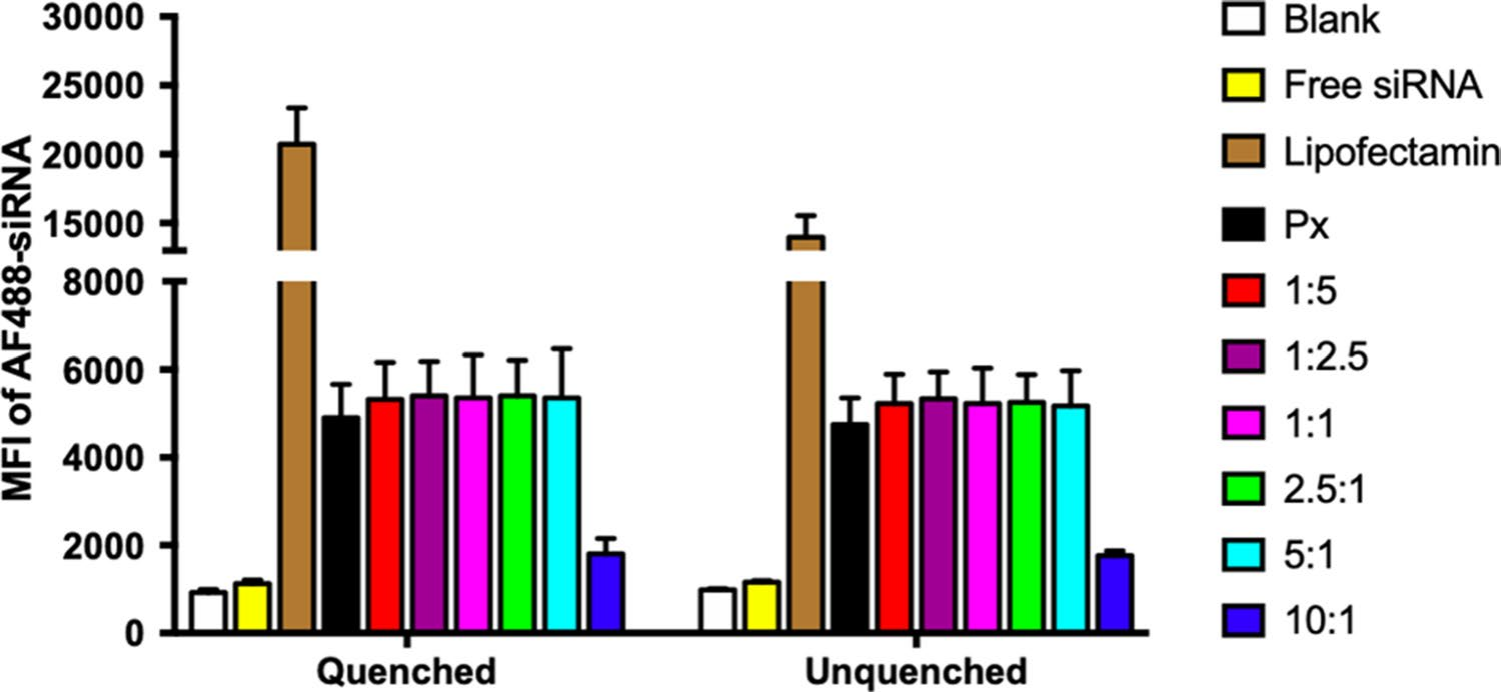
Cellular uptake of Alveofact-coated polyplexes in H1299 cells. Cellular uptake was evaluated after 24 h of transfection with polyplexes encapsulating 50 pmol of AF488-siRNA. Median fluorescence intensity (MFI) was determined by flow cytometry. Negative controls consisted of untreated cells and samples treated with free siRNA. Positive controls consisted of Lipofectamine2000 lipoplexes. Data points indicate mean ± SEM (*n* = 3).

To investigate the cellular internalization, a human lung epithelial cell line (H1299) was transfected with Alveofact-coated polyplexes at different Alveofact:PEI coating ratios encapsulating Alexa Fluor 488-labeled siRNA. The samples were analyzed by flow cytometry to obtain median fluorescence intensity (MFI) values of the transfected cells. Negative controls consisted of untreated cells as well as free AF488-siRNA, while positive controls consisted of Lipofectamine2000 lipoplexes. The experiment showed a slight improvement in MFI when increasing the Alveofact content for polyplex coating, approximately 10–20% higher in comparison to uncoated polyplexes, yet the differences were not significant. In this regard, De Backer *et al*. [19] reported the reduction in cellular uptake of Curosurf-coated siRNA-loaded nanogels in murine alveolar macrophage cell line due to the anionic pulmonary surfactant shell. Given that our coated polyplexes retained an overall positive charge, it can be deduced that the electrostatic interaction between coated polyplexes and cell membranes was not influenced by the presence of pulmonary surfactant shielding. Undeniably, an increased particle size hampered the internalization process, leading to a sharp drop in MFI at Alveofact:PEI ratio of 10:1. In addition, trypan blue quenching was performed to eliminate extracellular fluorescent signals resulting from siRNA bound to the cell membrane but not internalized by cells. The experiment resulted in no significant MFI differences between quenched and unquenched samples, confirming the cellular internalization of the different formulations tested (Fig. 3).

### *In Vitro* Transfection Efficacy in Lung Epithelial Cells

**Fig. 4:**
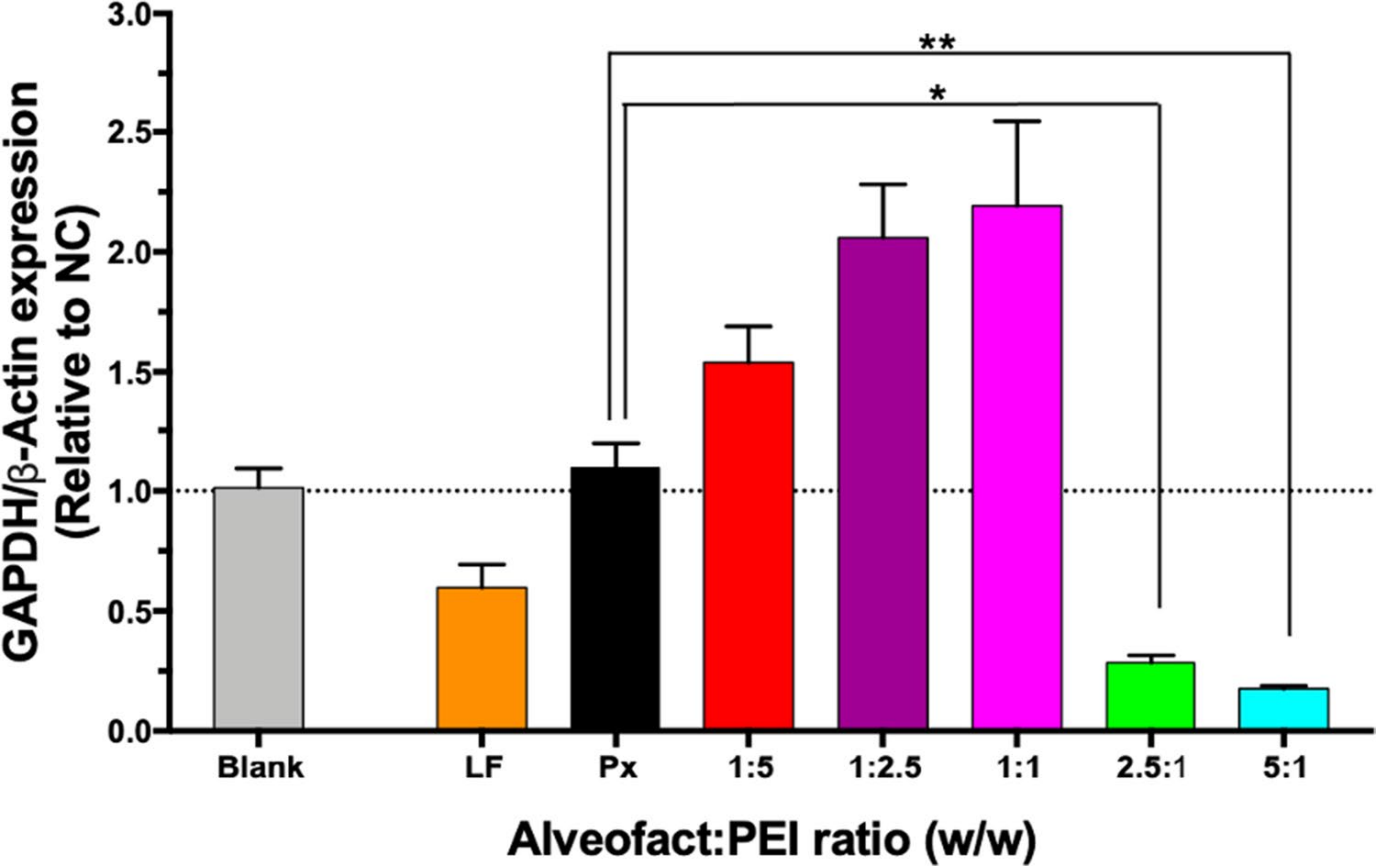
GAPDH gene knockdown of Alveofact coated polyplexes in 16HBE14o- cells. GAPDH gene knockdown efficiency was evaluated 24 h after transfection with polyplexes. Blank samples consisted of 16HBE14o- cells treated with HEPES 10 mM pH 7.4. Negative controls consisted of polyplexes encapsulating scrambled-sequence siRNA. Positive controls consisted of Lipofectamine2000 lipoplexes. GAPDH expression was normalized with β-Actin expression and quantified by qRT-PCR. Downregulation efficiency was displayed by the relative of GAPDH/β-Actin expression of targeting samples normalized to the GAPDH/β-Actin expression after treatment with negative control siRNA in the same formulation. Data points indicate mean ± SEM (n = 3). One way ANOVA, * *p* < 0.05, ** *p* < 0.01.

After confirming the cellular uptake of Alveofact-coated polyplexes, we further evaluated their ability of silencing the endogenously expressed housekeeping gene GAPDH in a more relevant lung epithelial cell line. We chose the human bronchial epithelial cells (16HBE14o-) as they more closely represent the main features of the pulmonary epithelium, particularly since they present tight junction properties, which play a critical role in the barrier of airway lining [34]. We anticipated that Alveofact coating might have an impact on tight junction proteins, namely Zonula occludens-1 (ZO-1) and occludin through hydrophobic and hydrophilic interaction [35, 36]. It is reported that phospholipid content of surfactant might increase epithelial permeability, thus opening tight junctions [37]. Furthermore, the proteins present in lung surfactant, like the hydrophobic SP-B and SP-C, play also an important role in increasing cytosolic delivery [38]. Consequently, the presence of lung surfactant could be beneficial for improving the internalization of our delivery system might, and siRNA could thereby reach the cytosol more efficiently. Indeed, as illustrated in Fig. 4, while Lipofectamine displayed about 41% GAPDH gene silencing, polyplexes at Alveofact:PEI ratios of 2.5:1 and 5:1 significantly mediated GAPDH gene silencing capacity of 72% and 83% respectively. Interestingly, low Alveofact content (Alveofact:PEI ratios of 1:5, 1:2.5, 1:1) did not improve the downregulation efficiency in comparison to uncoated polyplexes but increased GAPDH expression. Therefore, we can conclude that well defined concentrations of Alveofact coating improved the efficiency of the delivery system by mediating a significant downregulation of the target gene.

### *In Vitro* Cell Viability on Lung Epithelial Cells

**Fig. 5:**
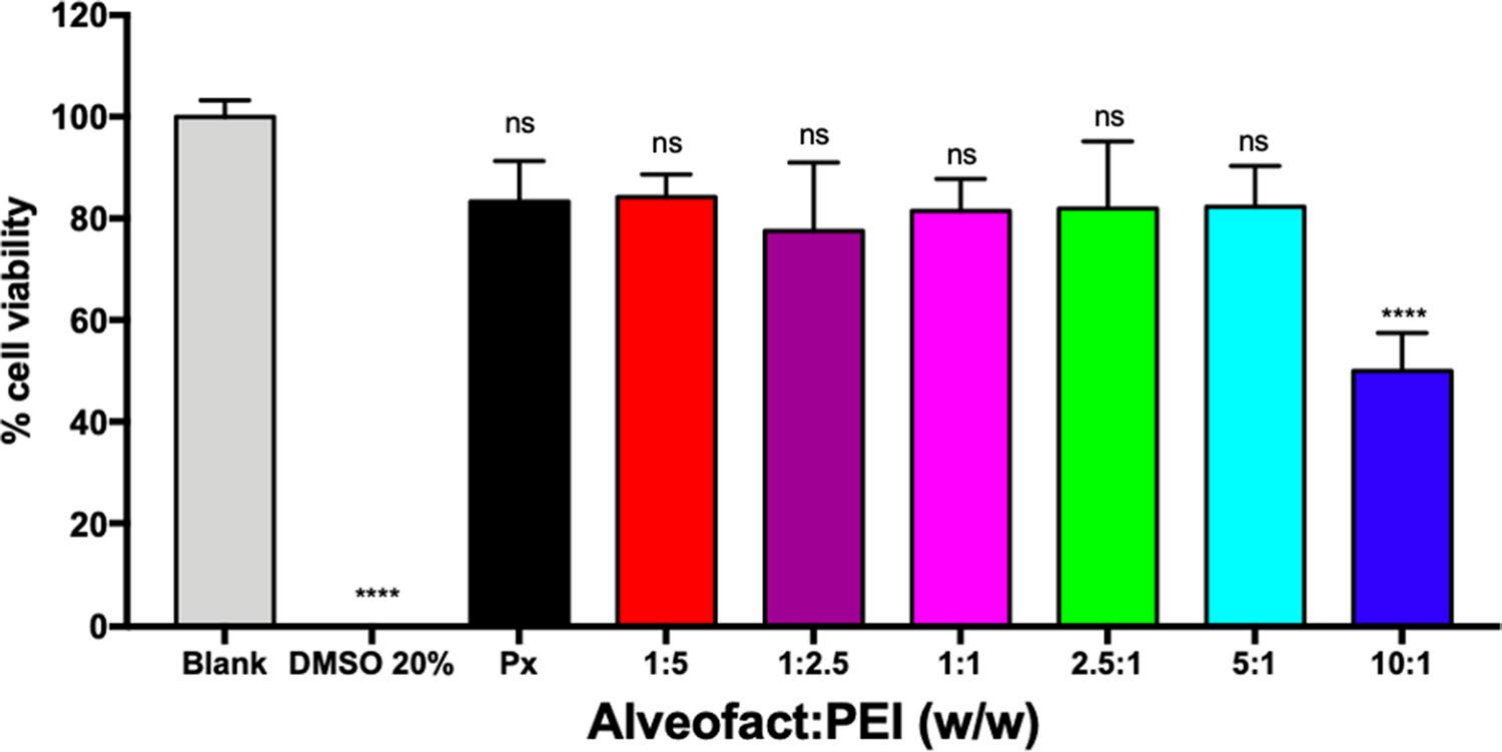
Evaluation of cell viability following the incubation of 16HBE14o- cells with Alveofact-coated polyplexes by MTT assay. 100% cell viability consisted of cells treated with HEPES 10 mM pH 7.4 buffer, while 0% cell viability consisted of cells treated with 20% DMSO. Data points indicate mean ± SEM (*n* = 3). One way ANOVA, ns = not significant, **** *p* < 0.001.

To test the compatibility of Alveofact coated polyplexes with lung epithelial cells, an MTT assay was performed after incubation with the polyplexes prepared at the different Alveofact:PEI ratios. Viable cells can metabolize the water-soluble MTT into formazan crystals, which serves as an indicator of cell viability [39]. Untreated cells and cells treated with 20% DMSO were assigned as 100% cell viability and 100% cell death, respectively. Figure 5 shows the results from the viability assay. All tested formulations showed an overall safe profile in comparison to the cells receiving no treatment. At 10:1 ratio, the large hydrodynamic diameter together with the high concentration of Alveofact, not only hampered the cellular uptake as described above, but also played a deleterious effect on cell growth, resulting in a significant reduction of cell viability. Nonetheless, the formulations with the best performance in terms of activity, 2.5:1 and 5:1 Alveofact:PEI ratio, showed safe profiles with about 85% cell viability.

### *In Vitro* Delivery of Alveofact-Coated Polyplexes to an Air-Liquid Interface Culture System

**Fig. 6:**
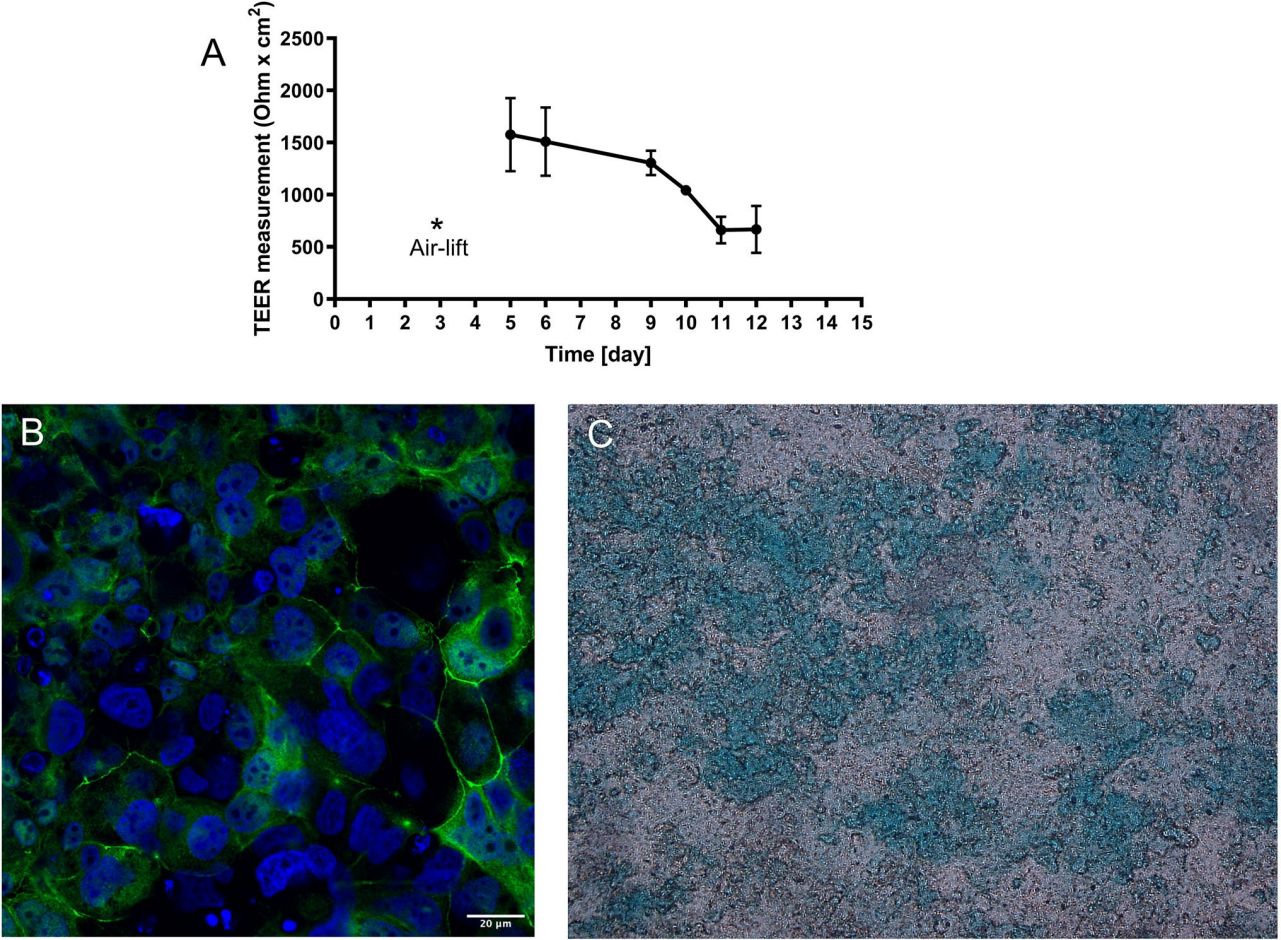
Characterization of 16HBE14o- cell line at the air-liquid interface. (**A**) TEER values of 16HBE14o- cells at ALI culture for 12 days. Cells were seeded onto Transwell at day 0, inserts were exposed to air (Air-lift) at day 3, and TEER values were measured from day 5. Data points indicate mean ± SD (*n* = 5). (**B**) ZO-1 staining of 16HBE14o- cells in ALI culture after 7 days of air-lift, bar = 20 μm. Green color corresponds to ZO-1 stained with rabbit ZO-1 antibody as primary antibody and AF488-anti-rabbit as secondary antibody (green), while nuclei were stained with DAPI (blue). (**C**) Mucus staining of 16HBE14o- cells in ALI culture after 7 days of air-lift. Blue color corresponds to mucus.

After confirming the activity of the Alveofact-coated polyplexes on a lung epithelial cell line, we evaluated their behavior in an experimental setup more closely resembling the *in vivo* environment typical of the airways. When considering pulmonary administration, it is indeed very important to establish an *in vitro* model that includes the hurdles found in the lungs, especially regarding the mucus barrier. In this view, air-liquid interface cultures represent a valid tool for recreating the main features of the respiratory tract *in vitro*. By exposing the cells to the culturing medium on one side and to the air on the other, they can form a pseudostratified epithelium with tight junctions between cells as well as secreting mucus [40]. Many studies have shown the suitability of ALI cultures as tools for mimicking healthy and diseased states of the lung, such as cystic fibrosis, asthma or viral infections [24]. The 16HBE14o- cell line is also suitable for ALI culture [41]. Therefore, we established an ALI 3D culture model with this cell line to test Alveofact-coated polyplexes in a more sophisticated environment. First, we confirmed the formation of the epithelial barrier by measuring TEER values. On day 2 after air-lift, TEER values as high as 1500 Ω*cm^2^ were observed, though slightly decreasing after 7 days (Fig. 6A). This phenomenon was already reported by previous studies in literature, suggesting that the decline in TEER values did not reflect a compromised cell layer barrier, but was rather caused by increased transcellular conductance [42]. Therefore, we confirmed the establishment of a stable epithelial cell layer suitable for further studies. The results were also supported by the expression of tight junctions between cells, as observed by confocal microscopy following ZO-1 staining (Fig. 6B). Furthermore, we confirmed the secretion of mucus 7 days after air-lift by alcian blue staining (Fig. 6C). By showing the development of high TEER values, tight junctions and mucus secretion, we confirmed the establishment of a 3D *in vitro* model suitable for further investigation of Alveofact-coated polyplexes.

**Fig. 7:**
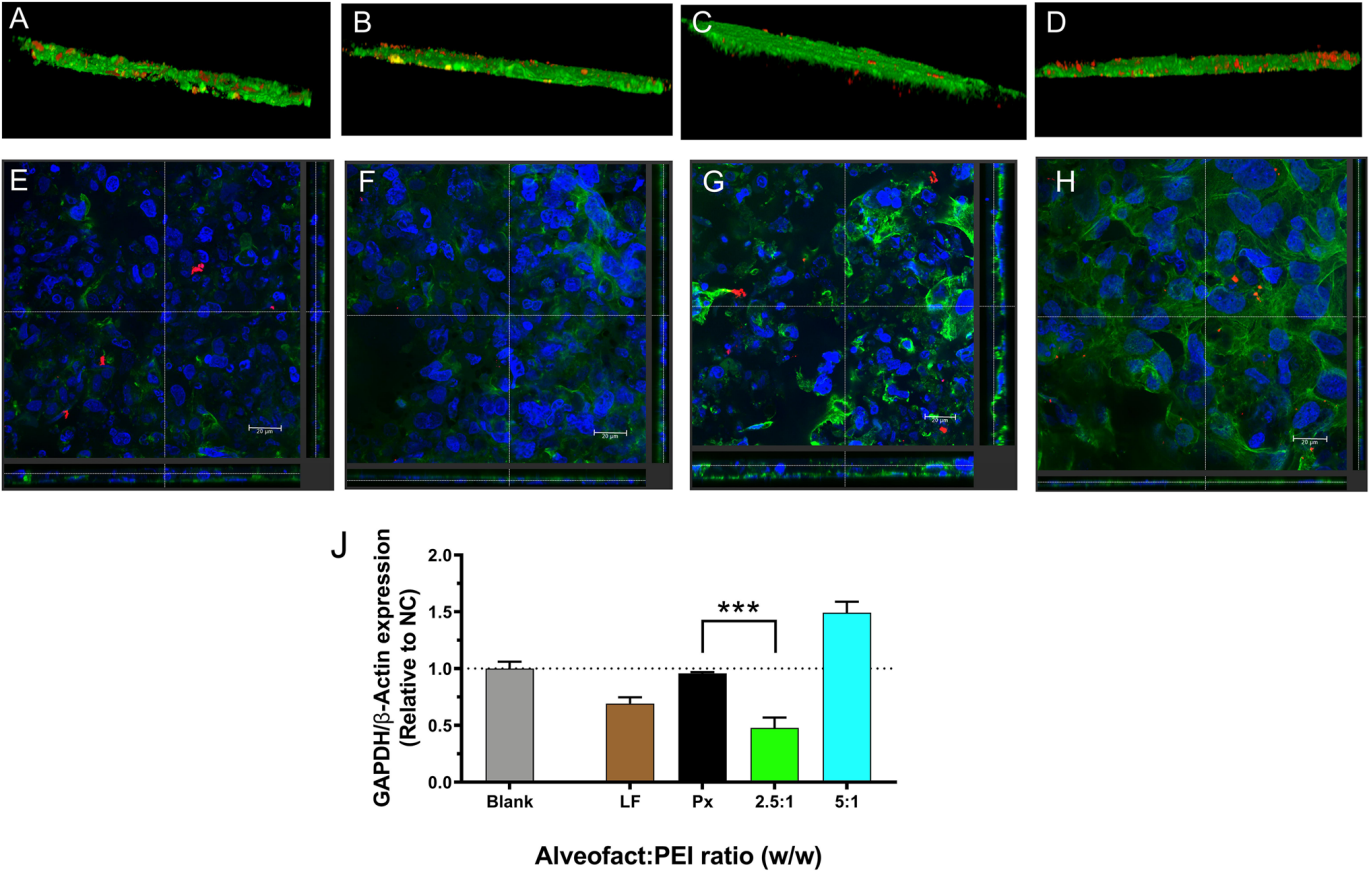
Evaluation of Alveofact-coated polyplexes on 16HBE14o- cells grown at air-liquid interface culture. (**A**, **B**, **C**, **D**) 3D construction of mucus penetration in 16HBE14o- cells 24 h after transfection with Lipofectamin2000 lipoplexes, uncoated polyplexes, Alveofact-coated polyplexes ratio of 2.5:1 and 5:1, respectively. Green represents mucus layer stained with AF488-labeled wheat germ agglutinin, red corresponds to AF647-siRNA. (**E**, **F**, **G**, **H**) Cellular uptake in 16HBE14o- cells 24 h after transfection with Lipofectamine2000 lipoplexes, uncoated polyplexes, Alveofact-coated polyplexes ratio of 2.5:1, and 5:1, respectively. Analysis was performed with confocal light scanning microscopy and images were presented in XY and XZ viewing direction, bar = 20 μm. Green corresponds to actin stained with rhodamine phalloidin, red to AF647-siRNA, and blue corresponds to nuclei stained with DAPI. (**J**) GAPDH gene knockdown efficiency of Alveofact coated polyplexes in 16HBE14o- cells grown in ALI culture 24 h after transfection with siGAPDH and scrambled siRNA as negative controls. Blank samples consisted of 16HBE14o- cells treated with HEPES 10 mM pH 7.4. Positive controls consisted of Lipofectamine2000 lipoplexes. GAPDH expression was normalized with β-Actin expression and quantified by qRT-PCR. Downregulation efficiency was displayed by the relative of GAPDH/β-Actin expression of targeting samples over negative controls. Data points indicate mean ± SEM (n = 3). One-way ANOVA, *** *p* < 0.005.

After the establishment of a 3D air-liquid interface culture of the lung epithelium, we evaluated the behavior of Alveofact-coated polyplexes in terms of cellular uptake, mucus penetration and transfection efficacy. Cell layers were transfected with the formulations showing the best performance in terms of activity in 2D culture (2.5:1 and 5:1 Alveofact:PEI coating ratio) as well as uncoated polyplexes and Lipofectamine2000 lipoplexes as controls. To test the ability of the polyplexes to diffuse through the mucus layer, polyplexes were loaded with a labelled AF647-siRNA, while mucus was stained with AF488-wheat germ agglutinin and the samples were analyzed at a confocal scanning laser microscope. In this study, Lipofectamine2000 lipoplexes as well as PEI polyplexes appeared to great extent trapped in the mucus mesh (Fig. 7A, B). On the other hand, 2.5:1 Alveofact-coated polyplexes (Fig. 7C), showed the best performance in terms of mucus diffusion. In fact, while the 5:1 ratio showed a partial entrapment in the mucus mesh similarly to the samples treated with lipofectamine and uncoated polyplexes (Fig. 7D), the ones treated with 2.5:1 ratio displayed only negligible entrapment in the mucus. To support these findings, a further staining was performed to better understand the fate of siRNA after crossing the mucus barrier. Here, nuclei (blue) and cytoskeleton (green) were stained, while AF647-siRNA is represented by red dots. In line with the previous results, the best cellular uptake was observed for 2.5:1 Alveofact:PEI coating ratio, followed by lipofectamine lipoplexes, which also reached the cells to some extent (Fig. 7E–G). On the contrary, almost no siRNA was detected in the cells after treatment with uncoated polyplexes and 5:1 Alveofact:PEI coated polyplexes (Fig. 7H).

After investigating the influence of the mucus layer on the delivery of siRNA to the cells, the consequences on the activity of the formulation were yet to be understood. Therefore, we transfected the cells with an siRNA sequence against GAPDH, as previously tested in 2D culture. Thus, we directly compared the impact of the cellular model on the activity of the formulation. As it can be observed in Fig. 7J, the results were in fact quite surprising. While the activity of uncoated polyplexes and Lipofectamine2000 lipoplexes were related to the one observed in the submerged culture, that was not the case for Alveofact-coated formulations. While the 2.5:1 coating retained its activity and achieved about 50% GAPDH downregulation, the 5:1 coating, which showed the best activity in the submerged culture, showed no activity at all. This result can be explained by comparing the activity results to the mucus diffusion study. While the 2.5:1 coating ratio showed an acceptable mucus penetration, the 5:1 formulation seemed to be almost completely entrapped in the mucus mesh, therefore explaining the complete loss of activity in the 3D culture model. The discrepancy observed between the results from ALI experiments and 2D culture (Supplementary Fig. 3) as well as in the physicochemical characterization can be explained by the fact that while in the latter the stability of polyplexes were tested in a more artificial and less sophisticated environment, in the former a more complex environment was established for the experiment. In this way, harsher conditions allowed to better define stability and gene silencing efficiency profiles of the different formulations. This study underlines the importance of adopting appropriate models for testing the activity of the formulations, which better predict the *in vivo* activity, such as air-liquid interface cultures [43]. Nonetheless, we identified a formulation with potential for pulmonary administration of siRNA, thanks to its improved mucus penetration activity as well as transfection efficacy in a relevant *in vitro* model closely resembling the respiratory tract.

## Conclusion

In this study, PEI polyplexes were coated with Alveofact, a commercially available pulmonary surfactant, to achieve a formulation for pulmonary administration of siRNA. The coating process was optimized to achieve a formulation with desirable physicochemical parameters and stability. Alveofact coated polyplexes efficiently delivered siRNA to lung epithelial cells and were well tolerated. Furthermore, an ALI culture of the lung epithelium was established and used to assess the behavior of the newly developed delivery system in a more sophisticated 3D cell culture model. From this study, we identified a formulation able to penetrate the mucus layer as well as to mediate an efficient gene silencing. In summary, these findings show that Alveofact coating of cationic polymers such as PEI represents an appealing strategy to improve the delivery of siRNA to the lungs. Coating with Alveofact could in fact improve two important aspects of PEI-mediated siRNA delivery, that are mucus diffusion and gene silencing activity. The combination of these two aspects led to an overall improved outcome in comparison to uncoated polyplexes, which reinforces the rationale behind using lung surfactant for drug delivery. In conclusion, this study confirms the potential of Alveofact-coated polyplexes for targeting lung epithelial cells and it offers a new formulation strategy for efficient siRNA delivery to the lung.

## Supplementary Information

ESM 1 (DOCX 188 kb)
