# Supplementary material for: Optimization of Lung Surfactant Coating of siRNA Polyplexes for Pulmonary Delivery
Source: Pharm Res. 2022 Nov 29;41(1):77–91. doi: 10.1007/s11095-022-03443-3 (PMC9708138; doi:10.1007/s11095-022-03443-3)
Supplement: Supplementary file 1 — (DOCX 188 kb) [file 11095_2022_3443_MOESM1_ESM.docx]

**SUPPLEMENTARY INFORMATION**

**
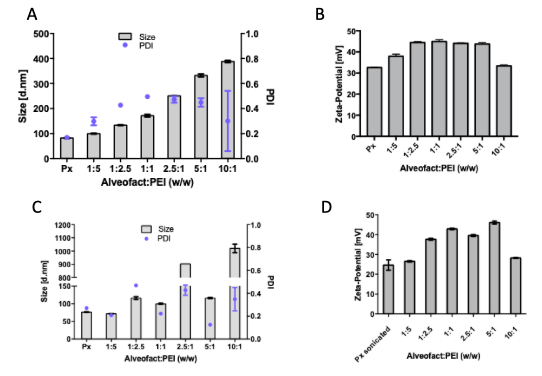
**

Supplementary Figure 1. Physico-chemical properties of Alveofact-coated polyplexes (A,B) without sonication and, (C,D) with a single Alveofact sonication step prior to incubation with siRNA/PEI polyplexes. (A,C) Hydrodynamic diameter and polydispersity index, and (B,D) ζ-potential of Alveofact-coated polyplexes prepared at N/P 6 in HEPES 10 mM pH 7.4 at different Alveofact:PEI coating ratios.

Supplementary Table 1. Evaluation of siRNA condensation efficiency of uncoated polyplexes and Alveofact-coated polyplexes at Alveofact:PEI ratios of 1:5, 1:2.5, 1:1, 2.5:1, 5:1, and 10:1 by SYBR Gold assay. Polyplexes encapsulate 100 pmol of scrambled siRNA in HEPES 10 mM pH 7.4. Free siRNA represents 100% siRNA release. Data points indicate mean ± SD (n=3)


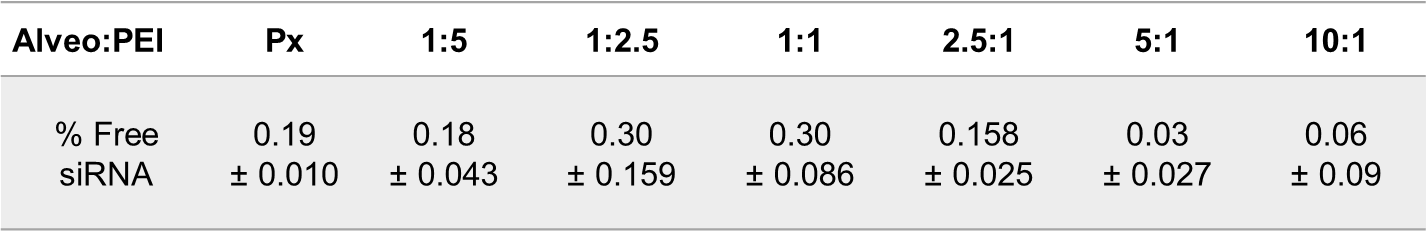


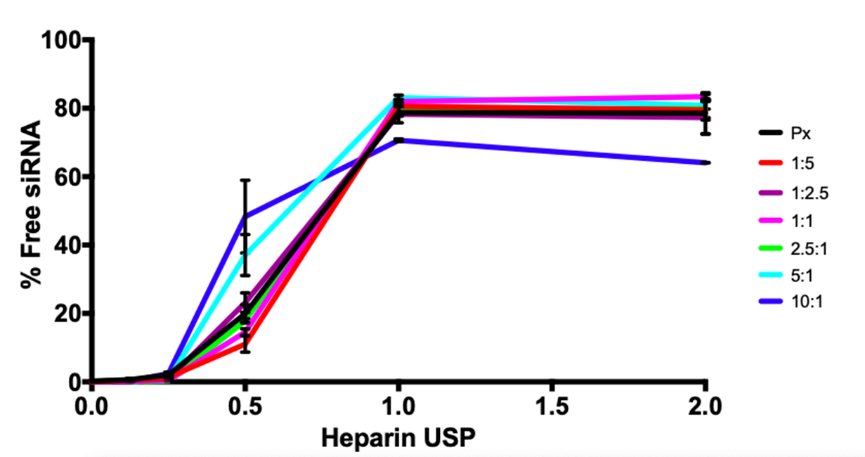


Supplementary Figure 2. Evaluation of siRNA release capacity of uncoated polyplexes and Alveofact-coated polyplexes in HEPES 10 mM pH 7.4 after 30 minutes of incubation with 0.125, 0.25, 0.5, 1, 2 USP units of Heparin by SYBR Gold assay. Free siRNA represents 100% siRNA release. Data points indicate mean ± SD (n=2).


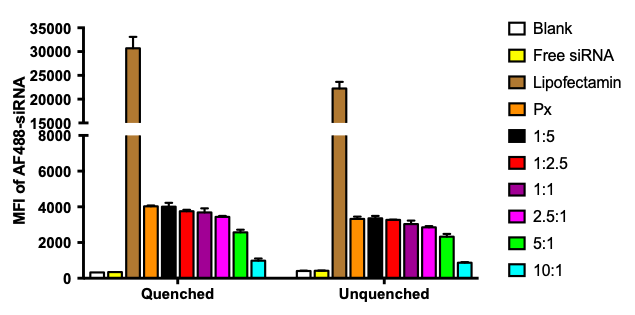


Supplementary Figure 3. Cellular uptake of Alveofact-coated polyplexes in HBEC in 2D culture. Cellular uptake was evaluated after 24 h of transfection with polyplexes encapsulating 50 pmol of AF488-siRNA.
